# Modelling the Epidemiological Impact of Intermittent Preventive Treatment against Malaria in Infants

**DOI:** 10.1371/journal.pone.0002661

**Published:** 2008-07-16

**Authors:** Amanda Ross, Melissa Penny, Nicolas Maire, Alain Studer, Ilona Carneiro, David Schellenberg, Brian Greenwood, Marcel Tanner, Thomas Smith

**Affiliations:** 1 Department of Public Health and Epidemiology, Swiss Tropical Institute, Basel, Switzerland; 2 Disease Control and Vector Biology Unit, Department of Infectious and Tropical Disease, London School of Hygiene and Tropical Medicine, London, United Kingdom; University of Oxford, United Kingdom

## Abstract

**Background:**

Trials of intermittent preventive treatment against malaria in infants (IPTi) using sulphadoxine-pyrimethamine (SP) have shown a positive, albeit variable, protective efficacy against clinical malaria episodes. The impact of IPTi in different epidemiological settings and over time is unknown and predictions are hampered by the lack of knowledge about how IPTi works. We investigated mechanisms proposed for the action of IPTi and made predictions of the likely impact on morbidity and mortality.

**Methods/Principal Findings:**

We used a comprehensive, individual-based, stochastic model of malaria epidemiology to simulate recently published trials of IPTi using SP with site-specific characteristics as inputs. This baseline model was then modified to represent hypotheses concerning the duration of action of SP, the temporal pattern of fevers caused by individual infections, potential benefits of avoiding fevers on immunity and the effect of sub-therapeutic levels of SP on parasite dynamics. The baseline model reproduced the pattern of results reasonably well. None of the models based on alternative hypotheses improved the fit between the model predictions and observed data. Predictions suggest that IPTi would have a beneficial effect across a range of transmission intensities. IPTi was predicted to avert a greater number of episodes where IPTi coverage was higher, the health system treatment coverage lower, and for drugs which were more efficacious and had longer prophylactic periods. The predicted cumulative benefits were proportionately slightly greater for severe malaria episodes and malaria-attributable mortality than for acute episodes in the settings modelled. Modest increased susceptibility was predicted between doses and following the last dose, but these were outweighed by the cumulative benefits. The impact on transmission intensity was negligible.

**Conclusions:**

The pattern of trial results can be accounted for by differences between the trial sites together with known features of malaria epidemiology and the action of SP. Predictions suggest that IPTi would have a beneficial impact across a variety of epidemiological settings.

## Introduction

Intermittent preventive treatment in infants (IPTi) involves giving antimalarial drugs at scheduled times during the first year of life, irrespective of whether the infants have malaria infections [1]. The limited number of doses is intended to retain the benefits of weekly or fortnightly chemoprophylaxis whilst avoiding the disadvantages: thus reducing malaria morbidity and mortality while minimising difficulties in sustainability, accelerating drug resistance or impairing the development of natural immunity.

IPTi trials to date have shown a strong, albeit variable, protective efficacy against clinical episodes of malaria in the first year of life [2]. How the impact of IPTi varies over time and in different epidemiological settings is unknown. Prediction is hampered by the lack of knowledge of both how IPTi works and the extent to which different trial characteristics may account for the variability in the observed estimates. Trial characteristics which have been highlighted are levels of drug resistance, transmission intensity, seasonality, IPTi schedule, and other interventions for malaria control (such as insecticide-treated nets (ITN) and treatment coverage) [2]–[4]. We use these characteristics as inputs to a stochastic simulation model of malaria epidemiology. We then modify this model to represent hypotheses that have been proposed for the mechanism of IPTi to investigate which of these hypotheses are consistent, and which cannot be reconciled, with the observed trial results. The hypotheses, defined in the Methods section, concern the duration of action of SP, the temporal pattern of fevers caused by individual infections, the potential benefits for acquired immunity of avoiding episodes and the effect of sub-therapeutic levels of SP on parasite dynamics. We then use the model which best fits our criteria to make predictions of the impact of IPTi in different epidemiological settings and with varying drug characteristics.

## Methods

### Model 1 (Baseline model): Model of malaria epidemiology taking into account between-trial differences

We combine a published model of malaria epidemiology [5] with an added component for the action of SP [6] and input the different trial characteristics such as transmission intensity and treatment coverage. This allows us to see if the between-trial differences in combination with this model can account for the heterogeneity in observed efficacy estimates.

#### Model for malaria epidemiology

**Figure 1:**
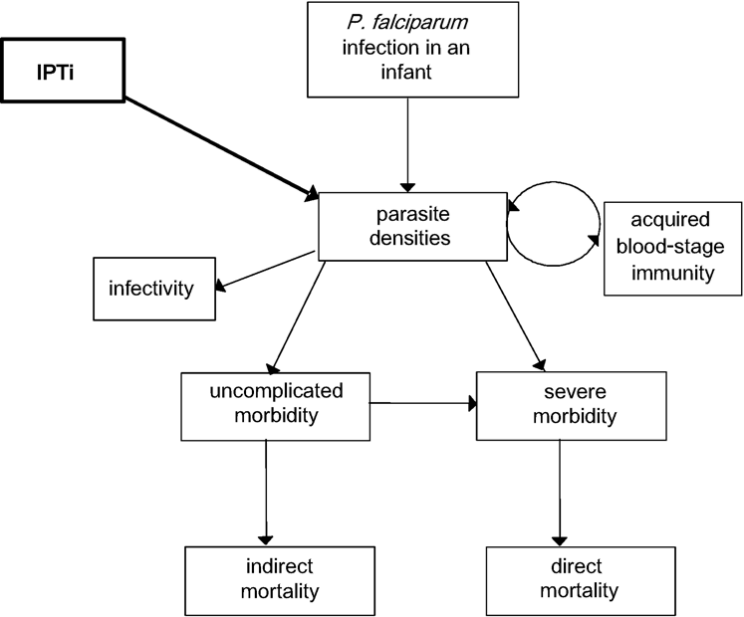
Simplified processes in the baseline model.

The model is individual-based and stochastic, and is fully described elsewhere [5]. Briefly, there is a simulated population of individuals who are updated at five-day timesteps via model components representing new infections, parasite densities, acquired immunity, morbidity, mortality and infectivity to mosquitoes (Figure 1). The course of parasite densities over an infection are described by averaged empirical data (described in [7]). Immunity to asexual parasites is derived from a combination of cumulative exposure to both inoculations and parasite densities, and maternal immunity [7]. The inclusion of acquired immunity allows us to model potential effects of IPTi on immunity through loss of exposure. The probability of a clinical attack of malaria depends on the current parasite density and a pyrogenic threshold (described in [8]). The pyrogenic threshold responds dynamically to recent parasite load, increasing or saturating through exposure to parasites and decaying with time, and thus is individual- and time- specific. Severe malaria can arise in two ways, either as a result of overwhelming parasite densities or through uncomplicated malaria with concurrent non-malaria co-morbidity [9]. Mortality can be either direct (following severe malaria) or indirect (uncomplicated malaria in conjunction with co-morbidity, or during the neonatal period as a result of maternal infection) [9]. The parameter values for this model were estimated by fitting to data from a total of 61 malaria field studies of various different aspects of malaria epidemiology, [10], [11] and are given in Table S1.

#### Simulation of sulphadoxine-pyrimethamine and drug resistance

The benefits of SP depend on a combination of the drug concentration and the frequency of mutations conferring drug resistance present in the population [6], however the exact time-course and killing action of SP is not known [12]. Hastings and Watkins quantify the chances of failing treatment with correct dosing for dihydrofolate reductase (*dhfr*) wildtype, 108, doubles, triples at 0, 0, 0, 50% respectively, while periods of preventive effect are 52, 12, 12, 2 days [6]. We simulate the action of SP according to these numbers rounded to the 5 day time steps used by the simulation model. Although *dhps* mutations have been isolated at the sites, they are not considered in this study.

#### Simulation of clinical episodes

The primary trial outcome was clinical episodes, defined as detected fever or history of fever together with parasitaemia, and infants were regarded as not at risk for the following 21 days [3]. In our simulations, only fevers presenting for treatment were counted as episodes and the infant was classified as not at risk for the following 4 five-day periods.

### Model 2: Alternative time duration for SP action

The duration of the prophylactic period for SP is not well established. We vary the duration of SP action from the baseline model, which has a prophylactic period of 50 days for wildtype infections [6], to 30 days. This alternative time period was chosen because drug concentrations of sulfadoxine and pyrimethamine alone decline log-linearly, but in combination they are synergistic and an isobologram suggests that there is a sharp drop in SP action after approximately a month [13]. Observations from field studies also suggest that the apparent effect of SP lasts for roughly one month [14]–[16]. Simulated infections are either sensitive or resistant, and the resistant infections are unaffected by drug treatment.

### Model 3: The timing of fevers produced by a single infection

**Figure 2:**
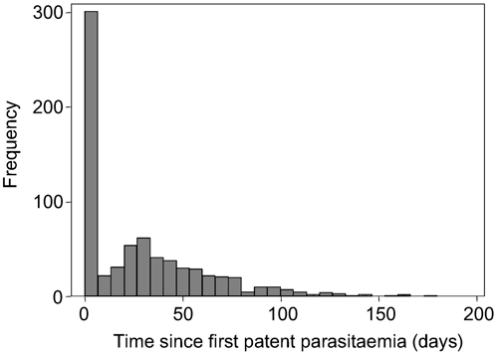
Timing of fevers* resulting from single infections in 334 neurosyphilis patients. *One fever counted per five-day interval. This data was collected by the United States Public Health Service in South Carolina and Georgia between 1940 and 1963 and was provided by Dr W Collins (Centers for Disease Control and Prevention, Atlanta, GA).

In non-immune adults inoculated with *P. falciparum* as treatment for neurosyphilis, untreated infections can persist for many months, during which clinical attacks recur at irregular intervals [17] (Figure 2). The infections cleared or prevented by IPTi would therefore have caused repeated fevers, some of which could have occurred 3 months or more after infection.

The timing of fevers is not well characterized by the baseline model which tends to produce too little variation, missing both early and late fevers. We therefore use an alternative, simple simulation model based on the empirical timing of fevers to examine whether the temporal pattern of fevers resulting from individual infections can account for the pattern of trial results for episodes.

Model 3 is different to the other models in that it is not based on model 1, other than the algorithms for the number of infections producing blood-stage parasites in each infant [18]. For each successful infection, we randomly selected one of the 334 malariatherapy patients' timing of fevers. Concurrent infections did not interact and there was no acquired immunity. SP was assumed to act in the same way as for the baseline model.

### Model 4: High parasite densities may not be efficient for acquiring immunity

Overwhelming parasite densities may not contribute as much to the accrual of immunity as would the same total number of parasites experienced in smaller doses over a longer period of time. Such densities cause fever, and the fever itself may also hinder the acquisition of immunity, possibly through the loss of T- and B-cells. We modify the baseline model to reduce the contribution of parasite density to acquired immunity in the presence of a fever.

In the baseline model (model 1), immunity is modelled as a function of both the number of distinct infections that the individual has experienced and his or her cumulative parasite load. The cumulative exposure to parasites for individual *i* of age *a* at time *t*, *X_y_*(*i*,*t*), is defined as the cumulative sum of daily densities of asexual parasites/microlitre of blood since birth up to time *t*. This can be partitioned into the cumulative sum up to time *t*-1, the previous five-day time-step, and the sum of the densities over the last five days, *Y*_5_(*i*,*t*),
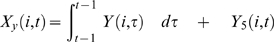



For model 4, we include a parameter *β_f_* which fixes the contribution of the current density as *Y*_5_(*i*,*t*) if a malarial fever is absent (*β_f_*_ = 0_ = 1), but may differ from this if a fever is present.
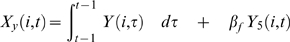



We fitted the new parameter *β_f_* to the same datasets used to fit model 1, simultaneously with the previously defined parameters [5], [10]. These parameters estimates are given in Table S1. To calculate *X_y_*(*i*,*j*,*t*) to correspond to the published model [7], we subtract the contribution of infection *j* to avoid double-counting.

### Model 5: Surviving infections are attenuated by SP allowing extended low-level exposure beneficial to stimulating immunity

Waning drug concentrations or partial drug resistance may allow parasites to survive in the presence of SP whilst restricting their growth [6]. This may allow an extended time for the immune system to mount a response to the parasite, which could facilitate the development of immunity to malaria. [19], [20]. It is not known if attenuated infections can lead to enhanced immunity in this way, although there is some experimental data from mice that suggests that this may be possible [21]. Low levels of blood stage infection in humans can induce immunity [22]. We hypothesize that infections beginning when SP concentrations have decreased to sub-therapeutic concentrations have reduced densities and longer durations compared to when there is no SP, and that this enhances the development of immunity.

We modify the baseline model so that a simulated infection beginning within a window period after SP treatment has a longer duration and lower densities. The window period begins as the prophylactic action ends, and the duration depends on the *dhfr* mutations assigned to the simulated infection (wildtype: 10 days; 108/double/triple: 30 days). We simulate parasite densities in the same way as for the baseline model, except that we reduce all densities from the infection by a third and extend the duration by a factor of 3. This value was arbitrarily chosen to represent an upper limit for plausible values. The potential consequence of model 5 is to increase the amount of time that an infant has low-level parasitaemia, which in turn increases the time that the pyrogenic threshold is high.

### Data sources: The field trials

A model of IPTi should capture the approximate time-course of efficacy of IPTi trials. The most detailed, standardised age-groups are those provided by a systematic analysis of six IPTi trials using SP [3]. For practical reasons, we omit studies not included in this report [23]–[26]. All six included trials were carefully conducted and independently monitored [3].

A critical input for the models is transmission intensity. Reported seasonal entomological inoculation rates (EIR) and/or age-prevalence curves were available from three trial sites (Manhiça, Ifakara and Navrongo) [15], [27]–[29], but not for the remaining three (Tamale, Kumasi and Lambaréné) [30]–[32]. Thus the formal comparison of models and empirical data was restricted to simulations from the former three trials, whilst the latter were used to validate model output against general patterns in the trial results.

For some age-groups, efficacy estimates for episodes defined with two different parasite density cut-offs are available: fever plus parasitaemia of any density, and fever plus high parasite density. In most cases there is little difference [3], [15], [27]–[29]. However, a discrepancy arises in Navrongo [3], [15] for children over one year of age where the estimated efficacy for high density episodes (≥5000/µl) suggests an increase in episodes in the IPTi group compared to the placebo group which is not apparent for episodes with parasitaemia of any density. In this case, we use the high density definition because it is likely to be more specific in a high transmission area and as age increases [33], [34].

### Specifying model input values for the trial sites

#### Transmission intensity, seasonality and ITN use

We based our model inputs on the published data for seasonality and transmission intensities (Table 1). In Ifakara, the extensive coverage of insecticide-treated nets (ITNs) may have substantially decreased transmission from the reported EIR of 30 per year. Our baseline model does not explicitly include a component for the impact of ITNs, this is currently being implemented [35]. However the most relevant consequence for modelling trials of IPTi would be the reduction in transmission to the infants. It is likely that ITN use would also decrease onward transmission, but is not expected to alter the sporozoite load of an infectious mosquito, nor would a lower sporozoite load be likely to lead to less severe outcomes in humans [36]–[38].

**Table 1:** Study sites and trial characteristics.

	Schellenberg *et al* [27], [28]	Chandramohan *et al* [15]	Macete *et al* [29]	Kobbe et al[31]	Mockenhaupt et al[32]	Grobusch et al[30]
Study site	Ifakara, Tanzania	Navrongo, Ghana	Manhiça, Mozambique	Kumasi, Ghana	Tamale, Ghana	Lambaréné, Gabon
Pattern of seasonality§	Perennial	Marked seasonality	Perennial	Perennial	Perennial	Perennial
Study period	1999–2001	2000–2004	2002–2005	2003–2005	2003–2005	2002–2006
Transmission Intensity (Infectious bites/adult/year)	29[61] in 1999–2000	418[62] in 2001–02	38 in 2001–02	approx 400	NK (high)	Approx 50
ITN coverage	67%	17%	0%	20%	<1%	5%
Untreated net coverage			15%	20%	<1%	85%
Day 14 ACPR* (95% CI)	66% (55,76)[63]	78% (69, 85)[64]	83% (73, 90)[65]	‡	86%(79,91)[66]	79% (64,90) † [67]
**Trial characteristics**						
Primary outcome: Protective efficacy first dose to 12 months[3]	58.8 (40.9, 71.3)	29.3 (17.3, 39.6)	20.1 (2.0, 34.9)	20.9 (8.9, 31.3)	33.3 (20.7, 43.9)	22.0 (−25.4, 51.5)
Number of infants enrolled (placebo/active)	351/350	1242/1243	755/748	535/535	600/600	595/594
Level of randomisation	Individual	Community	Individual	Individual	Individual	Individual
Schedule of IPTi doses (months)	2, 3 and 9	3, 4, 9 and 12	3, 4 and 9	3,9 and 15	3,9 and 15	3,9 and 15
Mean age at doses (months)	2.2, 3.3 and 9.2	3.0, 4.0, 9.5 and 12.6	3.3, 4.4, 9.4	2.8	2.4, 8.1, 14.3	3.1,9.3, 15.3
Coverage	100%, 95%, 84%	95%, 95%, 90%, 91%.	100%,96%,91%	100%,100%,99%	100%,98%,98%	
Method of case detection	Passive	Passive	Passive	Passive+Active (monthly)	Passive+Active (3-monthly)	Passive+Active (monthly)
First-line treatment	SP quinine	CQ and SP quinine	CQ or SP+AQ quinine	Artesunate+AQ	Artesunate	Artesunate (+AQ)
Rescue treatment						
Routine iron supplementation	Yes	Yes	No	No	No	No

* ACPR = adequate parasitological cure rates in clinical cases (6 months–5 years, or <5 years).

† Children aged 1–10 years.

ITN = insecticide treated net. NK = not know.

§ Roca-Feltrer *et al*, in prep.

‡ 79% infants with triple *dhfr* and/or *dhps* mutations at IPTi-3 [31].

For the Ifakara trial, we gauged the effective overall EIR by comparing observed age-prevalence [39], [40] and age-incidence curves for uncomplicated episodes [39] and malaria hospital admissions [41] to simulated age curves for a range of annual EIR values. The best-fitting age patterns were produced by an EIR of approximately 4. We also considered the effects of decreasing transmission intensity and reduced seasonality [41]. Decreasing transmission has been proposed as a possible explanation for the high protective efficacy estimates observed in the Ifakara trial [42]. For Manhiça and Navrongo, we did not adjust the overall EIR for ITN use. The inputs for the Manhiça field site have been previously characterized for the baseline model [43]. We validated our input EIR value for Navrongo by comparing the simulated age-prevalence curve against two sets of survey data [7], [44]. In addition, we restricted the simulations for Ifakara to infants who reached 2 months of age between August and April in order to correspond to the recruitment period.

#### Treatment of clinical episodes

Only simulated fevers presenting for treatment were counted as episodes and the infant was classified as not at risk for the following 4 five-day periods to correspond to the trial definitions [3]. The proportion of malaria fevers that presented for treatment in the trials is unknown. We estimated this proportion by assuming that fevers were treated with a constant probability. We adjusted this probability until our simulations of the time to first treated episode matched the published Kaplan-Meier curves for the placebo groups. The closest matches were found for Ifakara, Manhiça and Navrongo using 20%, 4% and 7% respectively. The value of 4% in infants in Manhiça was similar to the previous estimate of 5% for children 1–4 years in a vaccine trial [43]. The pattern of the estimates is plausible because the Ifakara study area was centred around a town with relatively good access to the health facility whereas the other trial settings were rural. We assumed that 48% of the severe episodes presented for treatment in all trials [9].

Clinical episodes presenting for treatment were given SP in Ifakara and SP with chloroquine (CQ) in Navrongo (Table 1). In Manhiça, the national policy changed from CQ to amodiaquine (AQ) plus SP during the course of the trial. We simulate CQ and SP as clearing all infections, sensitivity analyses show that this assumption is not critical. The rescue treatment in the Ifakara, Manhiça and Navrongo trials was quinine which was given if the infant was admitted to hospital with malaria, presented within 2 weeks of an IPTi or placebo dose, or presented within 14 days of receiving SP. We simulate quinine as clearing all infections within a five-day time-step.

#### Frequencies of dhfr mutations

Each simulated infection was assigned a genotype (*dhfr* wildtype, 108 or double mutations, or triple mutations). The frequency of *dhfr* mutations in each trial site is uncertain. Fourteen day adequate clinical and parasitological cure rates (ACPR) are available (Table 1), but they underestimate the true failure rate [12], [45]. It is not possible to determine by how much the rate is underestimated for an individual site because the 14 day parasitological failure rates have a low predictive value [45], [46]. Estimating *dhfr* genotype frequencies from data on the prevalence of mutations in infected humans is also difficult, because a combination of mutations may be formed in various ways when there are multiple infections. We aim to determine only whether the trial results can be reproduced for a reasonable assumed value of the frequency of *dhfr* mutations combined with the baseline model, and so we simulate the trials over a range of assumed frequencies. The lower bound of this range was provided by converting the lower confidence interval of the 14 day failure rates into *dhfr* genotype frequencies using simulations of the trials which had reported the 14 day failure rates. The value producing the best-fitting predictions within this range was chosen.

### Scenarios used for predicting the impact of IPTi outside of the trial settings

We predicted age-specific protective efficacy and cumulative protective efficacy up to the age of four years.

We define the cumulative protective efficacy as 
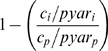
 where *c* is the cumulative number of episodes in the IPTi (*i*) or placebo (*p*) groups and *pyar* are the person-years at risk.

We also predicted the number of acute episodes, severe episodes and combined direct and indirect malaria deaths that would be averted for a period of 20 years following the introduction of IPTi in a population aged 0 to 90 years. We assumed a reference scenario with IPTi doses at 3, 4 and 9 months and then changed the values of different variables one by one to investigate their effects on the predicted impact (Table 2). The simulations were based on a population of 200,000 individuals, with an approximately stationary age-distribution matching that of the demographic surveillance site in Ifakara, Tanzania, in 1997–99 [47].

**Table 2:** Variables that vary between scenarios**.

Variable	Description	Levels
Intensity of transmission	Infected bites per adult per year prior to the introduction of IPTi‡	High transmission: 200
		Moderate transmission: 100
		**Reference: 21**
		Low transmission: 6
Treatment coverage	Proportion of malaria fevers treated	**4%**, 30%
Drug resistance	Frequency of 3 different genotypes	100%, 0%, 0%
		**80%**, **10%**, **10%**
		20%, 40%, 40%
		0%, 0%, 100%
Prophylactic period	Time in days that drug clears blood-stage infections for each of the 3 different genotypes †	0,0,0 days (treatment only)
		**50**, **10**, **0 days** (corresponds to SP)
		100, 20, 0 days
IPTi schedule	Age at doses	**3**, **4 and 9 months**
		Single doses 1.5–24 months*
IPTi coverage	Proportion of eligible infants receiving all 3 IPTi doses (coverage with first, second and third dose)	**89% (95%,95%,99%)**
		50% (79%,79%,79%)
		100% (100%,100%,100%)

** One variable was varied at a time. In each scenario, the variables not being evaluated were fixed at the reference levels (indicated in **bold**).

‡ The seasonality follows that of Namawala, Tanzania.[68] Each simulation assumes a recurring pattern of the vectoral capacity.

† The proportion of infections cleared by the genotypes are set at 100%,100% and 50%.

* We investigated the effect of age at dose by simulating a single IPTi dose at varying ages.

## Results

### Comparison of models with different mechanisms for IPTi

**Figure 3:**
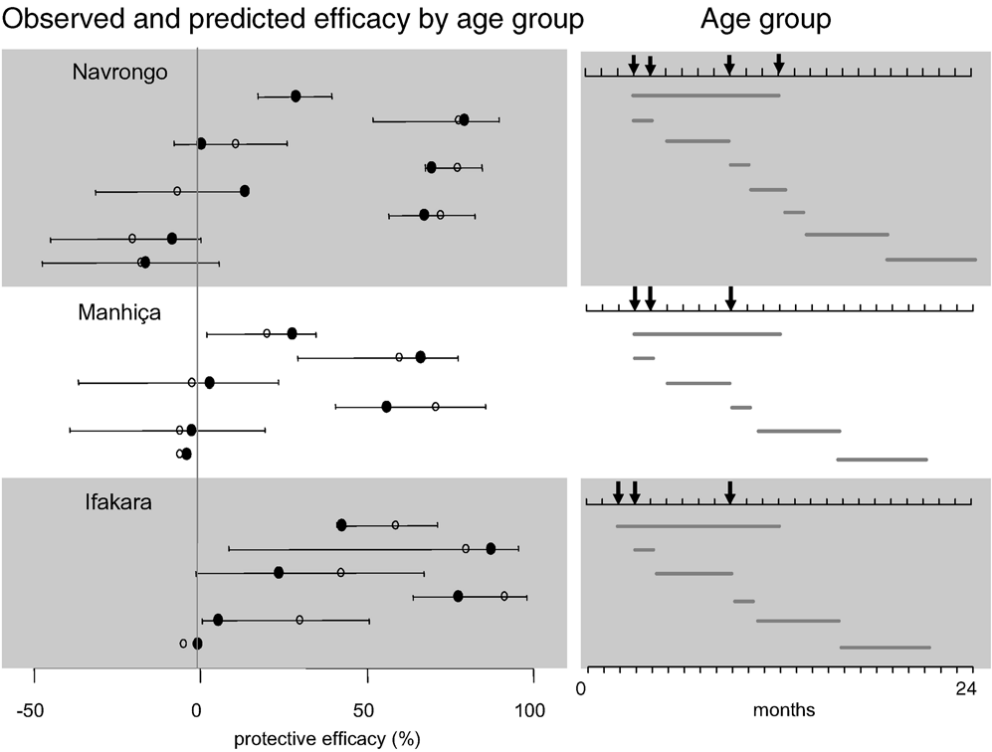
Comparison of trial estimates and baseline model predictions of protective efficacy of IPTi with SP against clinical episodes, by age group. Open circles = Trial estimate with 95% confidence interval. Filled circles = Baseline model prediction. Protective efficacy = percentage reduction in incidence of clinical episodes in IPTi group compared to placebo group. The age groups that the morbidity surveillance refers to are illustrated on the right-hand side. The arrows point to the scheduled ages at IPTi doses.

The agreement between the baseline model predictions and observed trial estimates was generally good (Figure 3 and Table 3). However, the continued positive protective effects of IPTi observed in Ifakara between doses and after the last dose were not fully captured. The Ifakara trial results for the periods between doses and after the last dose could be matched by reducing the transmission intensity as found in another study [42], but only if the intensity was reduced by at least 70% in the second year.

**Table 3:** Model fit for acute episodes assessed by weighted sums of squares.

	Description of model	Navrongo	Manhiça	Ifakara	Total
Model 1	Baseline	0.618	0.046	0.239	0.903
Model 2	30 day SP action	0.557	0.039	0.386	0.982
Model 3	Repeat episodes	1.515	0.534	0.089	2.138
Model 4	Avoiding fevers	0.699	0.043	0.180	0.922
Model 5	Attenuated infections	2.845	0.423	0.128	3.396

We calculated the squared difference between the trial estimate and the predicted protective efficacy, weighted them by the number of person years at risk/100 and summed them to give a measure of the goodness-of-fit. A smaller value indicates a better fit. The three trials with EIR measurements were formally used to test the models, the remaining three were used only to validate the model output against general patterns in the trial results.

We compared the fit of the different models using weighted sums of squares (Table 3). None of the alternative models substantially improved agreement over that of the baseline model. However, models 2 and 4 also produced predictions which fell within the confidence intervals of the estimates of protective efficacy obtained in the trials (not shown) and could not be ruled out as providing an explanation for the effects of IPTi. Altering the duration of SP action (model 2) improved the fit slightly in the case of Navrongo and Manhiça, but reduced the fit for Ifakara, in comparison to the baseline model. Overall, however, the predictions were similar to those of the baseline model which can most likely be attributed to the similarity of the assumed action of SP since only the duration was altered. The predictions made by model 4 (where fevers penalized the acquisition of immunity) did not substantially differ from those of model 1. The greatest difference was seen in the results for Ifakara, where the assumption of benefits to acquired immunity from avoiding fevers increased the predicted efficacy between doses and after the last dose. Models 3 and 5 both incorporated processes to lengthen the duration of SP action beyond the duration of active drug concentrations. Their results were not consistent with Navrongo trial estimates, since they failed to capture the lack of effect of IPTi between doses and after the last dose. However, these models best predicted the high efficacy estimates observed between doses and after the last dose in the Ifakara trial.

Model 1 also adequately predicted the impact of IPTi on hospital admissions (Table 4).

**Table 4:** Observed and predicted protective efficacy for severe episodes presenting for treatment.

	Observed hospital admissions with parasitaemia	Observed all-cause admissions	Predicted admissions due to severe malaria (model 1)
	**first dose-12months**		
Ifakara	58.5 (28.7, 75.8)	29.2 (6.6, 46.2)	48.7
Navrongo	50.2 (22.6, 68.0)	17.7 (−0.1, 32.3)	32.9
Manhiça	22.5 (−16.0, 48.2)	24.6 (7.2, 38.7)	30.0
	**5 months after last dose**		
Ifakara	15.3 (−65.0, 56.5)	−4.9 (−47.1, 25.2)	18.5
Navrongo	−14.2 (−95.9, 33.4)	−16.3 (−53.0, 11.6)	0.04
Manhiça	−32.0 (−114, 18.2)	8.1 (−25.7, 32.8)	−1.7

We also compared the output of the baseline model with the three trials not included in the formal comparison. They reported efficacy estimates in line with the Manhiça and Navrongo trials. Only previously unobserved features were used for further validation of the baseline model. In the trial in Kumasi [31], IPTi doses were given at 3, 9 and 15 months of age. Kumasi villages with higher incidence of malaria in the placebo group show a linear increase in observed protective efficacy in the following 6 months [48]. Model 1 did not reproduce this result. Our simulated protective efficacy showed either no change or a slight decrease over a wide range of incidence values. The observed association may be due to different health system coverage in the different villages [49], or the additional influence of increased acquired immunity on SP efficacy [48]. Alternatively, the observation may be due to different specificities of case definitions in the different villages [48]. The model would be able to capture the effect of treatment coverage if it is known, but at present is unable to capture the effect of increased immunity on SP action or effects of different specificities since non-malaria fevers are not modelled. Model 1 also did not fully capture the large negative efficacy observed for severe malarial anaemia in the post-intervention period in the trial in Tamale [32].

### Predicted impact of IPTi using the baseline model

**Figure 4:**
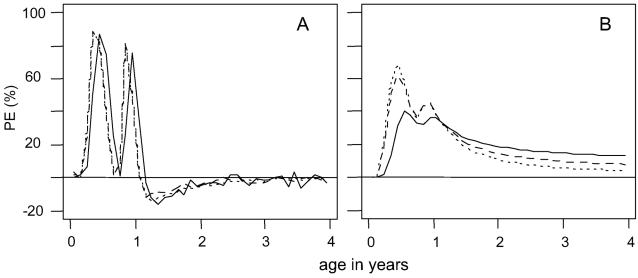
Predicted protective efficacy and cumulative protective efficacy by age. A. Predicted efficacy by age; B. Predicted cumulative efficacy by age for the reference scenario (summarized in Table 2) using model 1. IPTi doses were given following an EPI schedule at 3, 4 and 9 months of age. Dotted line = clinical malaria episodes; dashed line = severe episodes; solid line = malaria-attributable mortality.

Predicted patterns of protective efficacy by age were similar for acute malaria episodes, severe episodes and malaria-attributable mortality using model 1 (Figure 4a), with a small negative efficacy after the final dose for all outcomes. The slight delay in the peak protective efficacy for mortality is due to the inclusion of indirect malaria deaths, which occur as a result of an acute episode in conjunction with a co-morbidity and occur 30 days after the acute episode [9]. In contrast, the cumulative protective efficacy varied by outcome in the settings modelled (Figure 4b), at four years of age, the greatest effect was on mortality, followed by severe episodes. This is due partly to different predicted age-distributions of episodes in the placebo group and partly to age-dependent components in the model for severe episodes and mortality. The cumulative efficacy did not fall below zero for this or any of the other scenarios we have simulated.

**Figure 5:**
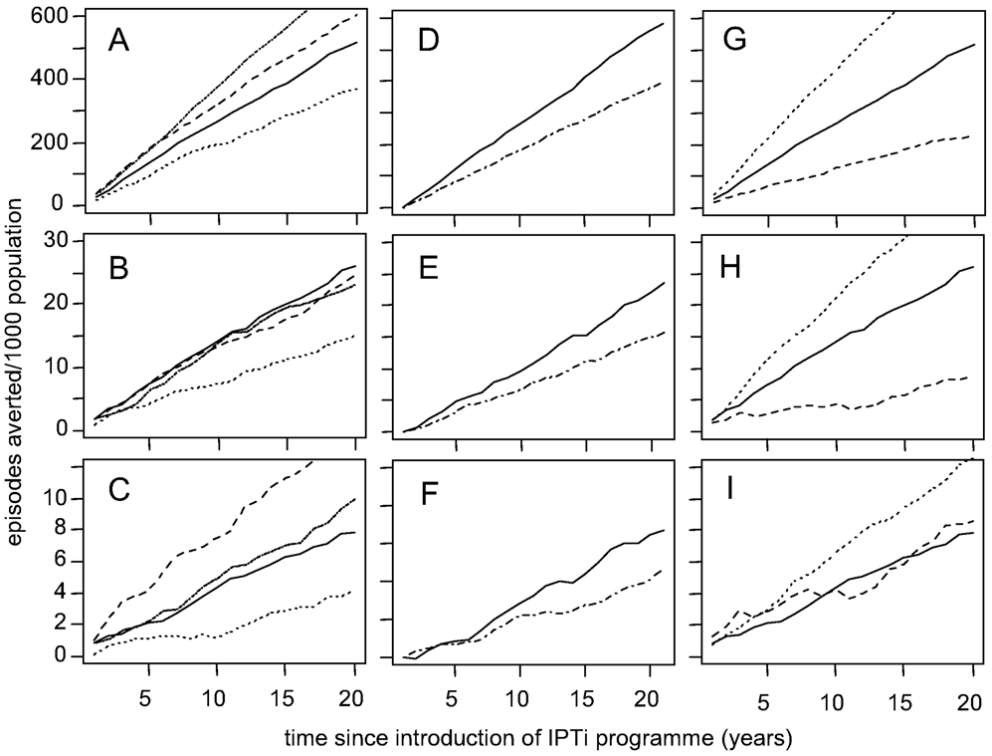
Predicted number of episodes averted by time since start of IPTi programme. First column: Effect of varying EIR from 6 (dotted line), 21(solid line), 100 (dash-dot line), 200 (dashed line) on A) clinical malaria episodes B) severe episodes, C) malaria-attributable mortality. Second column: Effect of varying health system coverage from 4% fevers treated (solid line) to 30% (dashed line) on D) clinical malaria episodes, E) severe episodes, F) malaria-attributable mortality. Third column: Effect of varying prophylactic period for sensitive infections from treatment effect only (dashed line), 50 days (solid line), 100 days (dotted line) on G) clinical malaria episodes, H) severe episodes, I) malaria-attributable mortality. Variables not being evaluated were fixed at the reference levels defined in Table 2.

The predicted number of episodes averted increased steadily over 20 years from the introduction of an IPTi programme (Figure 5). The linear increase reflects the negligible impact of IPTi on transmission and the short-term effects of IPTi in individuals. The predicted number of clinical episodes averted was greatest for moderate transmission settings (Figure 5a), but the number of deaths averted was greatest for higher transmission settings (Figure 5c). The number of deaths averted was greater for settings with a lower proportion of fevers treated and for IPTi drugs with a longer prophylactic period (Figure 5). Higher IPTi coverage and greater drug efficacy (or lower drug resistance) were also predicted to avert a greater number of episodes (not shown). The small predicted negative efficacy following the last dose as shown in Figure 4 was reduced both in settings where the impact of IPTi was less, such as with low drug efficacy or a high proportion of treated fevers, and in settings where there was low transmission intensity and thus little acquired immunity.

**Figure 6:**
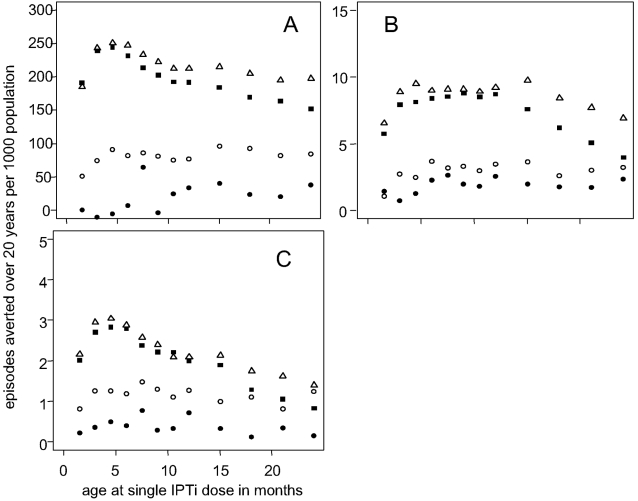
Episodes averted per 1000 population over 20 years by a single dose of IPTi by age at dose A) acute malaria episodes B) severe episodes C) malaria-attributable mortality. Filled squares EIR = 200; hollow triangles EIR = 21; hollow circles EIR = 6; filled circles EIR = 1.

We simulated the number of episodes averted for varying Expanded Programme on Immunization (EPI) schedules (not shown). Predictions suggested that the spacing of doses was important, with a greater number of episodes averted for doses at 4, 6, 9 months compared to 4, 5, 9 months. For simplicity, we show the effect of age at the time of doses by simulating a single dose although the number of episodes averted with a single dose is lower than with the three dose schedule. A single SP dose was predicted to have a beneficial impact for all of the transmission intensites and ages up to 24 months. The age at which the maximum number of acute episodes and deaths were averted for a single SP dose was approximately 5 months for both high and moderate transmission intensities, but there is no obvious peak within the first 24 months for low transmission intensities (Figure 6). For severe episodes, two peaks are apparent for the high and moderate transmission intensities. These reflect a shift between two types of severe malaria in the model. At younger ages, the majority of severe episodes averted are caused by an acute episode in conjunction with co-morbidity, and at older ages, overwhelming parasitaemia. For a single dose at older ages, the number of episodes averted by a single dose is greater for moderate transmission intensities. At low transmission intensities, it is been proposed that doses at later ages would avert the greatest number of episodes [50], and our predictions are consistent with this. However, there is greater uncertainty in our predictions for low transmission intensities due to the effects of heterogeneity [5], [10].

## Discussion

Trial-specific inputs together with the baseline model reproduced the pattern of trial results reasonably well. Although there was no clear ‘best model’, none of the alternative models substantially improved agreement. This indicates that known features of malaria epidemiology together with the duration of SP action can account for the trial results and the variability between them. However, other hypotheses involving interactions between drug concentrations and acquired immunity or fevers and acquired immunity could not be ruled out as possible mechanisms.

Predictions using the baseline model suggest that IPTi using SP is effective over a wide range of transmission intensities at reducing malaria clinical episodes and malaria-attributable mortality in infants. Small negative protective efficacy values were predicted for a short time following the prophylactic periods, but these were outweighed by the cumulative benefits.The predicted short-term impact of IPTi on an individual's level of immunity and negligible effect on transmission intensity produced a steady rate of cases averted in the community over time from the start of an IPTi programme. IPTi was predicted to avert a greater number of episodes where IPTi coverage was higher, the health system treatment coverage lower, and for drugs which were more efficacious. A greater number of episodes were also averted with longer drug prophylactic periods, agreeing with considerations that the prophylactic period is important for IPTi [51]. The predicted reductions in mortality were not as large as those observed with ITN programmes or interruption of transmission. IPTi has a similar effect on severe episodes as a pre-erythrocytic vaccine with assumed characteristics [52], but a much lower impact on uncomplicated episodes. This is likely to be due to the age-distribution of episodes and the longer-lasting effect of the pre-erythrocytic vaccine. The predictions also point to when IPTi is likely to not be useful. The number of cases averted is predicted to be fewer where IPTi coverage is lower, the health system treatment coverage is higher, and for short-acting drugs. At very low transmission intensities the predicted number of cases averted is few, however the model is likely to be less reliable at low transmission intensities [5], [10] and so it is not easy to determine a transmission intensity below which IPTi is not useful.

This study offers possible explanations for the very strong positive protective efficacy observed in the Ifakara trial between doses and following the last dose. Three models produced predictions consistent with the observed results (Table 3), two describing a process for the continued positive benefits of IPTi, either by enhancing the acquisition of immunity (model 5) or by clearing infections which may have caused future clinical episodes (model 3), and model 1 in conjunction with sharply decreasing transmission. Neither model 3 nor 5 reproduced the results from the other sites as well as model 1. In the case of model 5, it is not easy to see why enhancing immunity should work only at low transmission intensities. However, it is possible that effects resulting from the timing of episodes (model 3) are only apparent at low transmission intensities. They may be otherwise obscured by processes such as interactions between infections or acquired immunity. However, it is also possible that infections have shorter durations in infants than in adults [53]. Model 1 was able to reproduce the Ifakara trial results only if the initial EIR of 4 decreased by 70% or more in the second year. This is a substantial decrease and it is not known whether the transmission intensity did decrease so markedly over the study period (1999–2001). The incidence of uncomplicated episodes halved between 1995 and 2000 [41]. Decreasing transmission intensity was shown to be consistent with the Ifakara results in another modelling study, but also required a substantial decrease of 22% per month [42]. Although the causes underlying the Ifakara results remain unknown, it seems reasonable that transmission intensity, either low or decreasing, is likely to have played a role. The potential contribution of the high coverage of ITNs to the large impact of IPTi in Ifakara has been noted elsewhere [54].

The predicted impact on indirect malaria mortality was greater than that on direct malaria mortality. The predictions for indirect malaria mortality, and to a lesser extent, severe episodes rely on age-dependent co-morbidity functions. In a trial setting with access to good health care, the age-pattern of comorbidity may be quite different to that implicitly assumed by our models, which were fitted to other datasets [9]. In this case, the impact of IPTi on severe malaria and malaria-attributable mortality would be expected to be lower. Reductions in mortality have not been observed in the field trials reported to date, but the trials were not powered for this outcome.

This is the most comprehensive model to date, but still has certain limitations. The model predictions are unlikely to be reliable for low transmission intensities due to factors such as micro-heterogeneity and in-migration [10], and thus it is difficult to determine the range of transmission intenities where IPTi is not useful.

We were unable to capture the effects of drug levels on parasite population dynamics by the current within-host model which relies on empirical averaged parasite densities. A within-host model which will capture immune development more explicitly is in preparation, and will include several immune responses, fevers and antigenic variation. It will also allow a more realistic model of the action of SP.

The model component for the action of SP was compatible with our model of malaria epidemiology. It is a simple model derived from dose-response curves and isobolograms [6]. SP is assumed to act on the infection, either clearing it or not. This model would be unable to account for certain observed effects such as density-dependent cure rates or effects of acquired immunity. A more refined model would allow the drug concentrations to affect individual parasites. Such a model has been formulated by Gatton and colleagues [55]. All SP models to date have been constructed using data on SP concentrations in adults. There is evidence that SP is cleared more quickly in children and requires a greater dose per kilogram to reach the same concentrations [56], but little is known about infants. Data on the pharmacokinetics of SP in infants and the impact on infections with *dhfr* and *dhps* mutations are needed [57]. Adverse side-effects of SP are beyond the scope of this model. Although very rare, these have been reported [31]. The model also does not incoporate the effect of IPTi on levels of drug resistance, which has been modelled elsewhere [58], [59].

We did not include the impact of IPTi on anaemia in our model. Whilst anaemia is an important consequence of malaria, the lack of knowledge about the dynamic effects of malaria and anaemia on one another limits our ability to construct a satisfactory model. We have previously used a model relating anaemia to the population prevalence of parasitaemia [60] to predict the impact of pre-erythrocytic vaccines [52]. However, in the case of IPTi, the short-term blood-stage effects of the drugs and use of iron supplementation in some of the trials rendered this model unsuitable. A model of anaemia may be able to account for the severe malarial anaemia rebound which was observed in the trial in Tamale [32].

In conclusion, several models reproduced the trial data adequately so a single clearly preferred hypothesis for the secondary effect of IPTi on anti-malarial immunity cannot be identified. The previously published model adopted as our baseline model [5], with additional components for the action of SP, can reproduce the trial results using known features of malaria epidemiology. We propose that this model is suitable for making predictions of the impact of IPTi. These predictions suggest that IPTi would have a beneficial impact across a wide range of settings. These analyses contribute to a growing database of the likely effectiveness of different malaria control strategies generated using this common simulation platform [10].

## Supporting Information

Table S1 Parameter estimates for the models
(0.15 MB DOC)
