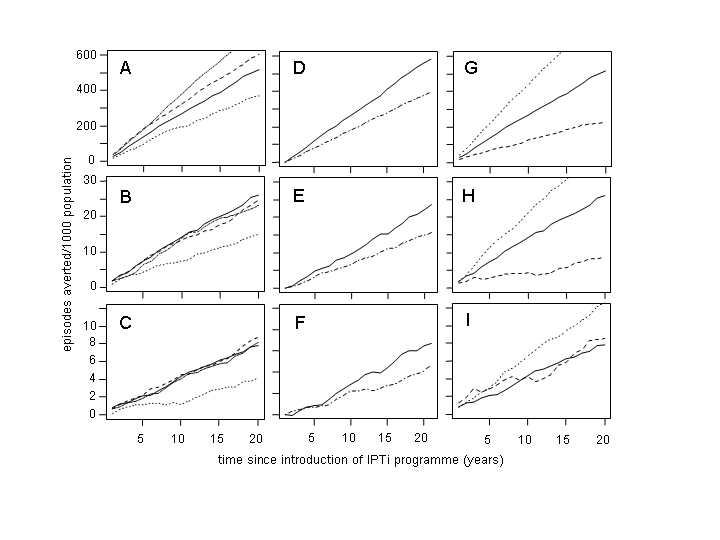# Correction: Modelling the Epidemiological Impact of Intermittent Preventive Treatment against Malaria in Infants

**DOI:** 10.1371/annotation/5dba0336-1efe-4387-8d9f-946b231331f3

**Published:** 2009-03-25

**Authors:** Amanda Ross, Melissa Penny, Nicolas Maire, Alain Studer, Ilona Carneiro, David Schellenberg, Brian Greenwood, Marcel Tanner, Thomas Smith

There is an error in Figure 5C. The dotted line for the predicted number of deaths averted in a setting with an EIR of 200 should reach 8.8 at 20 years. All other lines are correct. The correct figure can be viewed here: 

**Figure:**