# Supplementary material for: Modelling the Epidemiological Impact of Intermittent Preventive Treatment against Malaria in Infants
Source: PLoS One. 2008 Jul 16;3(7):e2661. doi: 10.1371/journal.pone.0002661 (PMC2441827; doi:10.1371/journal.pone.0002661)
Supplement: Table S1 — Parameter estimates for the models (0.15 MB DOC) [file pone.0002661.s001.doc]

Table S1. Parameter values used for models 1,2,4 and 5

| **Parameter** | **Description** | **Units/ dimension** | **Values for model 1^‡^** | **Values**  **for model 4** |
| --- | --- | --- | --- | --- |
| $S_{\infty}$ | Lower limit of success probability of inoculations at high $E_{a}(i,t)$ | Proportion | 0.049 | 0.049 |
| $E^{*}$ | Critical value of $E_{a}(i,t)$ | Inoculations/ person-night | 0.032 | 0.032 |
| $S_{imm}$ | Lower limit of success probability of inoculations in immune individuals | Proportion | 0.14 | 0.14 |
| $\gamma_{p}$ | Steepness of relationship between success of inoculation and$X_{p}(i,t)$ | Dimensionless constant | 2.06 | 2.04 |
| $X_{p}^{*}$ | Critical value of cumulative number of entomological inoculations | Inoculations | 2801.5 | 2116.3 |
| $X_{h}^{*}$ | Critical value of cumulative number of infections | Infections | 97.8 | 94.8 |
| $X_{y}^{*}$ | Critical value of cumulative number of parasite days | Parasite-days/μl x 10^-7^ | 13.8 | 6.5 |
| $X_{v}^{*}$ | Critical value of cumulative number of infections for variance in parasite densities | Infections | 0.92 | 0.92 |
| $a_{m}^{*}$ | Decay of maternal protection | Per year | 2.59 | 2.51 |
| $D_{x}$ | Effect of concurrent co-infections | Infections | 0 | 0 |
| $\beta_{1}$ | Effect of asexual density (lag 10 days) on expected gametocytaemia (fixed) | Dimensionless | 1 | 1 |
| $\beta_{2}$ | Effect of asexual density (lag 15 days) on expected gametocytaemia | Dimensionless | 0.46 | 0.46 |
| $\beta_{3}$ | Effect of asexual density (lag 20 days) on expected gametocytaemia | Dimensionless | 0.17 | 0.17 |
| $\rho$ | Location parameter for the distribution of the ratio of gametocytes to asexual parasites | Dimensionless | 0.00031 | 0.00031 |
| $\eta$ | Scale factor for probability that a mosquito becomes infected at any feed | Dimensionless | 0.56 | 0.56 |
| $\sigma_{g}$ | Standard deviation of the distribution of the ratio of gametocytes to asexual parasites | Dimensionless | 3.91 | 3.91 |
| $\alpha$ | Factor determining increase in $Y^{*}(i,t)$ | Parasites^2^μl^-2^day^-1^ | 157,000 | 179,000 |
| $\varpi$ | Decay rate of pyrogenic threshold | Year^-1^ | 2.5 | 2.5 |
| $Y_{0}^{*}$ | Pyrogenic threshold at birth | Parasites/μl | 328.1 | 244.3 |
| $Y_{1}^{*}$ | Critical value of parasite density in determining increase in $Y^{*}$ | Parasites/μl | 0.60 | 0.59 |
| $Y_{2}^{*}$ | Critical value of $Y^{*}(i,t)$ in determining increase in $Y^{*}(i,t)$ | Parasites/μl | 6502.3 | 6502.3 |
| $\beta_{f}$ | Contribution of five day parasitaemia to acquired immunity in the presence of a fever | Proportion | - | 0.80 |
| $Y_{B_{1}}^{*}$ | Parasitemia threshold for severe episodes type B_1_ | Parasites/μl | 347,000 | 258,000 |
| $F_{0}$ | Prevalence of co-morbidity/susceptibility at birth relevant to severe episodes (B_2_) | Proportion | 0.099 | 0.094 |
| $a_{F}^{*}$ | Critical age for co-morbidity | Years | 0.116 | 0.119 |
| $\phi_{1}$ | Case fatality for severe episodes in the community compared to hospital | Odds ratio | 2.07 | 2.09 |
| $Q_{n}$ | Non-malaria intercept for infant mortality rate | Deaths/1000 livebirths | 50.6 | 52.0 |
| $Q_{D}$ | Co-morbidity intercept relevant to indirect mortality | Proportion | 0.018 | 0.017 |
| $x_{MG}^{*}$ | Critical value of the simulated prevalence for ages 20-25 years | Proportion | 0.19 | 0.19 |
| $\mu_{\max}$ | Upper limit of risk of neonatal mortality in primigravidae | Proportion | 0.011 | 0.011 |
| $x_{PG}^{*}$ | Critical value of prevalence for neonatal mortality risk | Proportion | 0.25 | 0.25 |
| $\beta_{0}$ | Intercept | Log odds | -6.13 | -6.13 |
| $\beta_{P}$ | Effect of parasite prevalence | Log odds | 12.5 | 12.5 |
| $p*$ | Critical value of parasite prevalence | Proportion | 2.84 | 2.84 |
| $\beta_{a1}$ | Magnitude of age effect | Per year | 3.14 | 3.14 |
| $a*$ | Critical age | Years | 3.66 | 3.66 |
| $\beta_{I}$ | Age-prevalence interaction effect | Log odds | -0.75 | -0.75 |

**^‡^** Models 2 and 5 used the values of model 1.

The equations of the model are described elsewhere [5] as is a full description of the fitting process [10]. The parameter values were estimated simultaneously using distributed computing via the internet. They were harvested on 8 August 2007 from malariacontrol.net.
